# Designing Drugs for Parasitic Diseases of the Developing World

**DOI:** 10.1371/journal.pmed.0020210

**Published:** 2005-08-30

**Authors:** James H McKerrow

## Abstract

McKerrow outlines three new strategies, all originating within academic centers, that provide a new drug pipeline for treating parasitic diseases.

The term “parasite” derives from a classical Greek word that was used to refer to “a guest who comes to dinner and doesn't leave” or “a class of priests who had meals at public expense” (Clitodemus, in Athenaeus Grammaticus, ca. 378 B.C.). In modern usage, it refers to eukaryotic organisms that range from single-cell protozoa to complex multicellular worms. The diseases caused by these organisms represent some of the world's greatest health problems.

Malaria, for example, currently affects about 500 million people and causes about 3,000 deaths a day—mostly in sub-Saharan Africa [1]. Schistosomiasis, caused by Schistosoma spp. (blood flukes), affects over 250 million people in the tropical world [2], and a recent meta-analysis showed that the disease is significantly associated with anemia, chronic pain, diarrhea, exercise intolerance, and undernutrition [3]. Almost a billion people are infected with the nematode parasites: Ascaris (roundworm), Ancylostoma (hookworm), and Trichuris (whipworm) [2]. Why then have so few effective drugs been produced against these diseases?

The answer lies primarily in the fact that these are diseases affecting people who are poor and living in poor regions of the world. They represent little or no viable market for the pharmaceutical industry, especially given the market requirements of the recently merged pharmaceutical giants. Given this reality, how can imaginative and effective strategies be developed to meet this challenge?

## New Strategies for Antiparasitic Drug Design

At last year's meeting of the American Society of Tropical Medicine and Hygiene, the organization that hosts one of the largest international gatherings of basic scientists and tropical medicine specialists, several symposia highlighted efforts in antiparasitic drug design. What was five years ago a fairly dark vision of the future, now appears brighter.

Several nonprofit organizations are now operating that are dedicated to this unmet medical need, some collaborative interest exists in industry, and philanthropies have backed new initiatives. There are also a number of new academic consortia with novel strategies to address issues of target discovery and preclinical development.

**Strategy one: Developing a drug that has both a commercial market in the west and an application against a neglected parasitic disease.** This strategy is exemplified by the international consortium of researchers organized by Dr. Richard Tidwell of the University of North Carolina. With funding from the Bill and Melinda Gates Foundation, more than a dozen faculty and scientists from six research institutions (see Box 1) are working in collaboration with Immtech International to manage the “cross-over” development of antifungals produced by Immtech as potential drugs for African trypanosomiasis, also known as African sleeping sickness. This approach takes advantage of drug development of a class of compounds targeting a viable commercial market for Immtech (fungal diseases of the developed world). At the same time, academic- and nonprofit-institute scientists are targeting these compounds in parallel toward an “unprofitable” major health problem—African sleeping sickness. Specifically, an analog of the antifungal pentamidine has been optimized as a trypanocidal agent and is in clinical trials in Africa.

**Box 1. International Consortium Working with Immtech International to Develop Drugs for African Sleeping Sickness**

- University of North Carolina–Chapel Hill
- Georgia State University
- London School of Hygiene and Tropical Medicine
- Ohio State University
- Swiss Tropical Institute
- Kenya Trypanosomiasis Research Institute

**Strategy two: An academic consortium gets federal support for pharmacokinetic and toxicology studies.** This second strategy is exemplified by work under the leadership of Dr. Donald Krogstad and colleagues at Tulane University. Here, without an industrial partner, Krogstad and colleagues are using in-house chemistry and computational assistance to modify chloroquine analogs in an effort to overcome the problem of chloroquine resistance in malaria treatment. Their main partner in this effort is the National Institute of Allergy and Infectious Diseases, which has provided support for pharmacokinetic and toxicology studies by virtue of pre-existing contracts with SRI International (an independent nonprofit research-and-development organization) in Menlo Park, California, United States. At least one compound from this series is entering clinical trials.

**Strategy three: An academic center uses a philanthropic gift to woo expertise from industry and build infrastructure for preclinical drug development.** This third strategy is represented by the center that I direct, the Sandler Center for Basic Research in Parasitic Diseases at the University of California, San Francisco (UCSF). Here, philanthropic support was used to build infrastructure for a consortium of laboratories that mimics what might be found in a small- to medium-sized pharmaceutical company organized to carry out preclinical drug discovery and development. These core laboratories include computational support for drug discovery and chemical library selection, X-ray crystallography, drug metabolism and toxicology facilities, animal models of parasitic diseases, high-throughput screening, and synthetic chemistry.

Initial work at this center focused on a drug lead for Chagas disease and was supported by a Tropical Disease Research Unit Program project grant from the National Institute of Allergy and Infectious Diseases. This compound, now a drug candidate, was originally synthesized by Dr. Jim Palmer of Khepri Pharmaceuticals (now Celera) as part of an industry search for anti-inflammatory and anticancer drugs focusing on cysteine proteases. Validation of this compound as an antiparasitic was carried out by the UCSF team through rodent models of disease and, next, supported, as in the case of the Tulane consortium, by Dr. Chuck Litterst, Director of the National Institute of Allergy and Infectious Diseases Drug Development and Surveillance Group. The UCSF group initially handed over further development of a drug candidate to the Institute for OneWorld Health (iOWH), one of the nonprofit organizations that has sprung up to meet “downstream” drug development needs for neglected diseases (see Sidebar). iOWH facilitated tests of drug safety, minimum effective dose, and a formulation for large-scale Good Manufacturing Practice manufacture. The Drugs for Neglected Diseases Initiative (www.dndi.org) has now become the main partner for further development [4]. The Sandler Center consortium itself is now focusing on completing preclinical development of similar drug candidates targeting homologous enzymes in several other major global pathogens.

**Institute for OneWorld Health**

iOWH (www.oneworldhealth.org) is a nonprofit pharmaceutical company founded in 2000 in San Francisco whose mission is to develop safe, effective, and affordable new medicines for people with infectious diseases in the developing world. The institute carries basic research forward through drug development—it identifies drug leads, secures resources, and completes their development. For example, the organization recently received a grant of nearly US$10 million from the Bill and Melinda Gates Foundation to continue advancing its promising drug for visceral leishmaniasis, paromomycin, through the approval and post-approval process.

There is another way in which this third strategy represents a radical departure from the usual research paradigm of academic centers. A High-Throughput Screening Center has been established by the Sandler Center consortium, building upon the expertise of Drs. Kip Guy (now director of the center) and Janice Williams (now manager of the center), who brought an industrial perspective to the university. A recently completed screen used an assay that allowed robotic screening of a library of thousands of FDA-approved drugs and natural products for activity against Trypanosoma brucei, the parasite that causes African sleeping sickness. The “hit list” from this screen is being made available on a Web site (http://itsa.ucsf.edu/~schisto/fruit.html) for those organizations (such as the Drugs for Neglected Diseases Initiative [4], iOWH, and the World Health Organization) whose missions include licensing, manufacture, and distribution. Similar screens are being set up for leishmaniasis, Chagas disease, schistosomiasis, and malaria.

## A New Drug Pipeline

**Figure 1:**
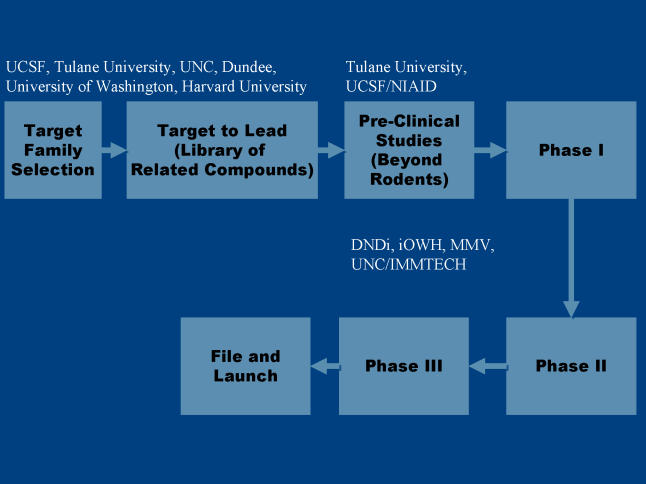
The Pipeline of Drug Development The success of the three strategies discussed in this article, all originating within academic centers, provides a new drug pipeline for parasitic diseases with no market value for the pharmaceutical industry. DNDi, Drugs for Neglected Diseases Initiative; IMMTECH, Immtech International; MMV, Medicines for Malaria Venture; NIAID, National Institute of Allergy and Infectious Diseases; UNC, University of North Carolina–Chapel Hill.

The success of these three unusual strategies, all originating within academic centers, provides a new drug pipeline for parasitic diseases with no market value for the pharmaceutical industry (Figure 1). The future is likely to see a number of academic or academic–industrial collaborations supporting preclinical development in consortia like those described above.

This prediction was already borne out at the April 2005 meeting, “Drug Development for Diseases of Protozoa,” sponsored by the Keystone Symposia. In the short time since the American Society of Tropical Medicine and Hygiene meeting, it has become clear that more academic laboratories are encouraged to pursue drug discovery and development avenues beyond research traditionally thought of as academic. New consortia modeled after the three described above are now functional or in late planning stages at the University of Washington, Johns Hopkins University, Harvard University, and the University of Dundee. In addition, significant help from some large pharmaceutical companies, notably the Trés Cantos laboratory of GlaxoSmithKline, has fueled focused academic- and nonprofit-organization drug development efforts. Three nonprofit organizations (the Drugs for Neglected Diseases Initiative, iOWH, and the Medicines for Malaria Venture) are actively working to support various stages of the drug-development pipeline. Their efforts are particularly key to the “downstream” elements of drug manufacture and clinical trials.

Finally, philanthropic organizations such as the Bill and Melinda Gates Foundation, the Sandler Family Supporting Foundation, and the Ellison Foundation have contributed to these endeavors. Their continued support, and the recruitment of other interested parties, will be crucial to maintaining the momentum engendered by the three projects outlined here.

*Editorial Note: PloS has received financial support in the form of a grant from the Sandler Family Supporting Foundation.*

## Abbreviations

iOWH: Institute for OneWorld Health
UCSF: University of California at San Francisco
